# Supplementary material for: Host Plants for the Lanternfly Saiva formosana Kato, 1929 (Hemiptera, Fulgoridae) Endemic to Taiwan, and Parasitism of Its Eggs by Wasps
Source: Insects. 2024 Oct 26;15(11):841. doi: 10.3390/insects15110841 (PMC11594280; doi:10.3390/insects15110841)
Supplement: Supplementary file 1 [file insects-15-00841-s001.zip › insects-3213252-supplementary.pdf]

Table S1. Checklist of *Saiva formosana* on different plants species.

**family name**	**species name**	**Publication date**
Aquifoliaceae	*Ilex asprella* Champ. ex Benth.	1852
Aquifoliaceae	*Ilex ficoidea* Hemsl.	1886
Aquifoliaceae	*Ilex micrococca* Maxim.	1881
Aquifoliaceae	*Ilex uraiensis* Yamam.	1932
Araliaceae	*Heptapleurum heptaphyllum* (L.) Y.F.Deng	2018
Cannabaceae	*Trema orientalis* (L.) Blume	1856
Daphniphyllaceae	*Daphniphyllum glaucescens* Blume	1827
Ebenaceae	*Diospyros eriantha* Champ. ex Benth.	1852
Elaeocarpaceae	*Elaeocarpus decipiens* F.B.Forbes & Hemsl.	1886
Euphorbiaceae	*Macaranga tanarius* (L.) Müll. Arg.	1866
Euphorbiaceae	*Mallotus paniculatus* (Lam.) Müll.Arg.	1865
Euphorbiaceae	*Triadica cochinchinensis* Lour.	1790
Euphorbiaceae	*Triadica sebifera* (L.) Small	1913
Fabaceae	*Acacia confusa* Merr.	1910
Fagaceae	*Quercus glauca* Thunb.	1784
Hydrangeaceae	*Hydrangea chinensis* Maxim.	1867
Lamiaceae	*Callicarpa formosana* Rolfe	1882
Lamiaceae	*Premna serratifolia* L.	1771
Lauraceae	*Machilus thunbergii* Siebold & Zucc.	1846
Lauraceae	*Machilus zuihoensis* Hayata	1911
Magnoliaceae	*Magnolia compressa* Maxim.	1872
Moraceae	*Ficus ampelos* Burm.f.	1768
Moraceae	*Ficus fistulosa* Reinw. ex Blume	1825
Moraceae	*Morus australis* Poir.	1797
Pentaphylacaceae	*Cleyera japonica* Thunb.	1783
Phyllanthaceae	*Glochidion rubrum* Blume	1826
Phyllanthaceae	*Glochidion zeylanicum* (Gaertn.) A. Juss.	1824
Primulaceae	*Ardisia sieboldii* Miq.	1867
Rosaceae	*Prunus persica* (L.) Batsch	1801
Rubiaceae	*Randia cochinchinensis* (Lour.) Merr.	1935
Rubiaceae	*Wendlandia formosana* Cowan	1932
Rutaceae	*Citrus maxima* (Burm.) Merr.	1917

Rutaceae	*Tetradium glabrifolium* (Champ. ex Benth.) T.G. Hartley	1981
Rutaceae	*Zanthoxylum ailanthoides* Sieb. & Zucc.	1845
Sapindaceae	*Acer serrulatum*	1911
Staphyleaceae	*Turpinia formosana* Nakai	1924

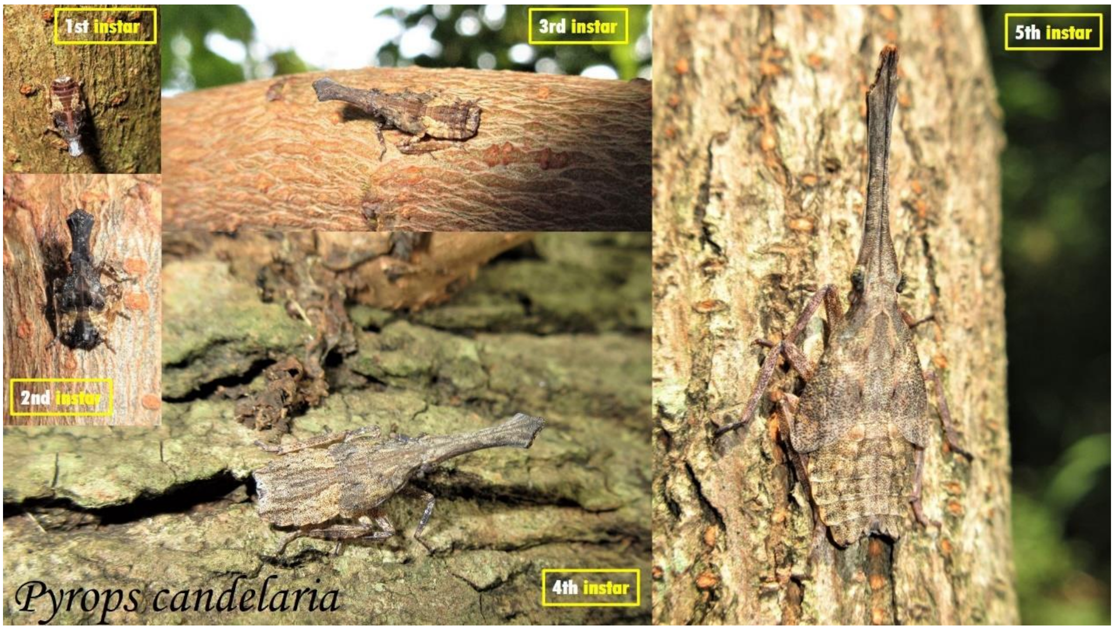

Figure S1. Dorsal views of *Pyrops candelaria* nymphs in various instars.

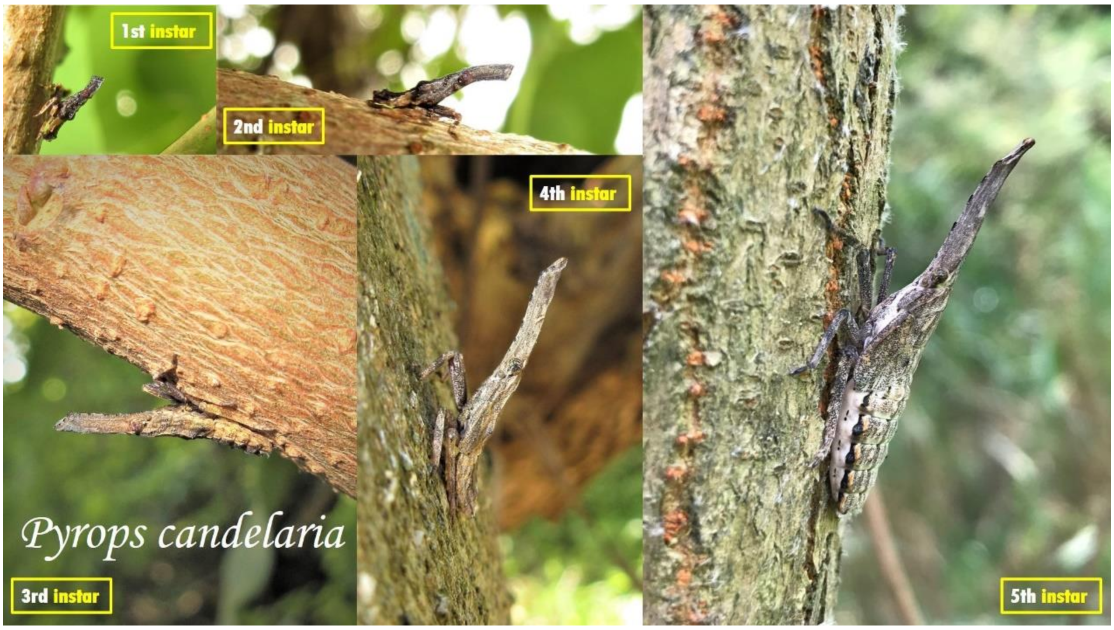

Figure S2. Lateral views of *Pyrops candelaria* nymphs in various instars.

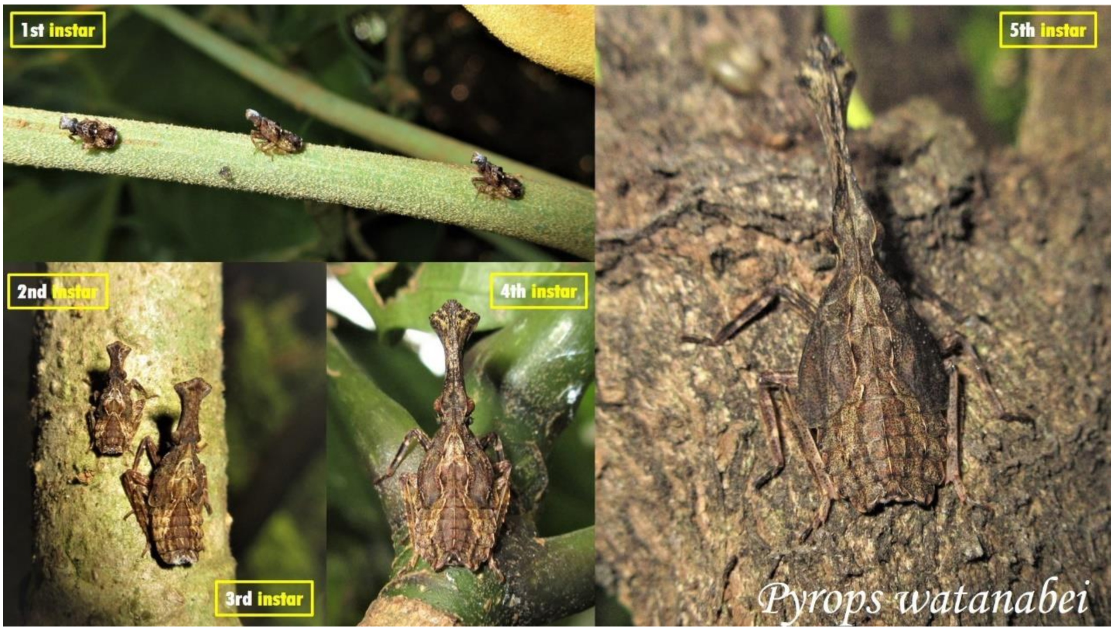

Figure S3. Dorsal views of *Pyrops watanabei* nymphs in different instars.

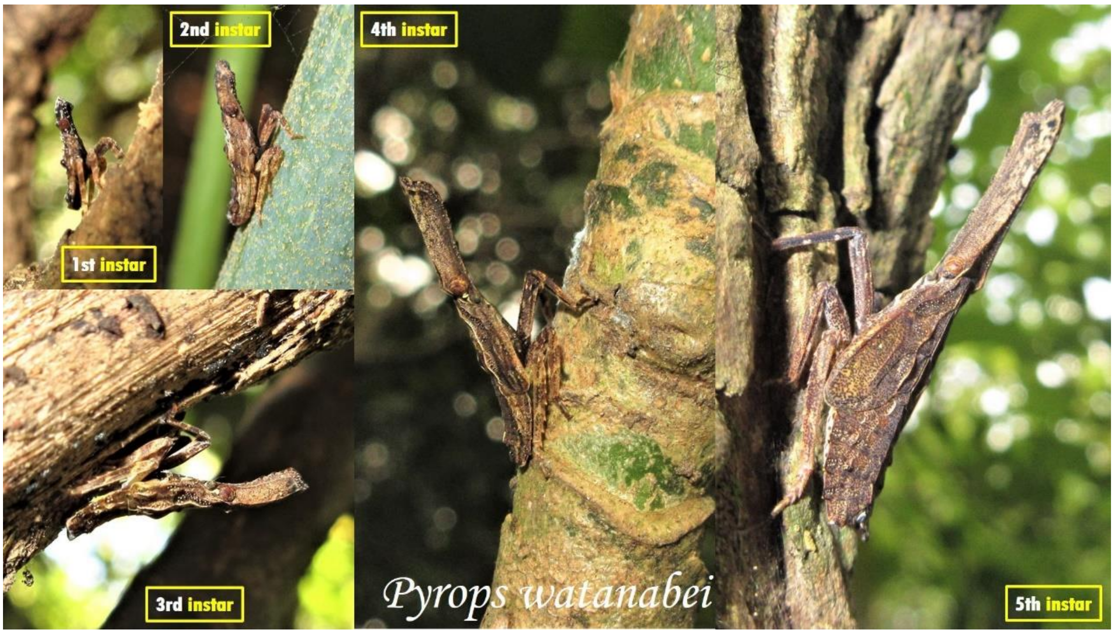

Figure S4. Lateral views of *Pyrops watanabei* nymphs indifferent instars.

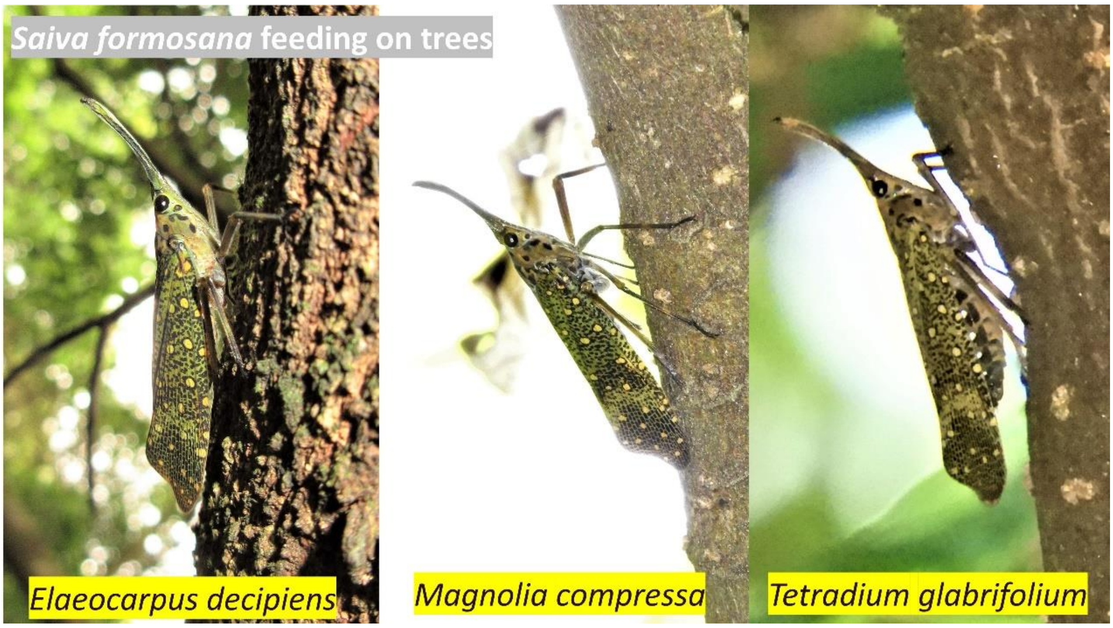

Figure S5. Adult *Saiva formosana* feeding on various trees.
